# Relationship Between Conventional Medicine Chapters in ICD-10 and Kampo Pattern Diagnosis: A Cross-Sectional Study

**DOI:** 10.3389/fphar.2021.751403

**Published:** 2021-12-20

**Authors:** Xuefeng Wu, Thomas K. Le, Ayako Maeda-Minami, Tetsuhiro Yoshino, Yuko Horiba, Masaru Mimura, Kenji Watanabe

**Affiliations:** ^1^ Center for Kampo Medicine, Keio University School of Medicine, Tokyo, Japan; ^2^ School of Medicine, Johns Hopkins University, Baltimore, MD, United States; ^3^ Division of Pharmaceutical Care Sciences, Graduate School of Pharmacy, Keio University, Minato-ku, Japan

**Keywords:** kampo, pattern diagnosis, international classification of diseases, traditional Japanese medicine, conventional medicine, ICD-10, ICD-11

## Abstract

**Objectives:** The newest revision to the International Classification of Diseases, the 11^th^ edition (ICD-11) includes disease classifications from East Asian medicine, including traditional Japanese medicine (Kampo medicine). These disease classifications allow for comparisons between disease classifications from conventional medicine and Kampo medicine.

**Design/Location/Subjects/Interventions:** This is an exploratory, cross-sectional study exploring the relationship between conventional medicine diagnoses and Kampo medicine diagnoses at a large Kampo clinic in Japan. Patients were seen from October 1st, 2014 to June 30th, 2019 and were 20 years of age or older.

**Outcome measures:** Patients presented with one or more conventional medicine ICD-10 codes into the clinic and were given one descriptor from the ICD-11 within the heat-cold module, excess-deficiency module, and an optional body constituents module. The distribution of these Kampo medicine codes was examined in relation to conventional medicine chapters.

**Results:** 1,209 patients were included in our final analysis. Patient number, ages, sex ratio, and BMI varied within conventional medicine ICD-10 chapters and Kampo medicine descriptor codes. Certain conventional medicine chapters are related to specific Kampo medicine descriptor codes, such as chapter IV (endocrine, nutritional, and metabolic diseases) with excess, heat, and kidney qi deficiency.

**Conclusion:** The advent of the ICD-11 allows for systematic, standardized comparisons between Kampo medicine, and contemporary medicine. In this exploratory study, our findings support the independence of Kampo medicine pattern descriptors with ICD-10 conventional medicine chapters. Code overrepresentations in relation to conventional medicine diseases and by age and sex should be an area of future investigation to best understand how to synergize and improve patient care.

## Introduction

Traditional Japanese medicine (Kampo medicine) is widely used in modern Japanese society. Physicians, regardless of specialty, prescribe Kampo in daily practice as standalone treatment or alongside conventional medicine (CM) (Moschik et al., 2012). In particular, Kampo formulas have been found helpful in specific diseases, such as pediatric emotional and behavioral disorders and *hiesho* (cold disorder) (Watanabe et al., 2014). Research on Kampo medicine has been increasing in recent years (Hyun et al., 2019). One important concept in Kampo methodology is *hosho sotai* (formula versus pattern). In other words, Kampo medicine does not employ the treatment based on a disease name but rather uses treatment on a set number of presenting symptoms, or a “pattern” (Yakubo et al., 2014).

In the International Classification of Diseases (ICD-11) traditional medicine (TM) chapter, patterns are defined as “the complete clinical presentation of the patient at a given moment in time including all findings” (WHO., 2019). Different Asian traditional medicine modalities use their own subset codes in their respective practices. In Kampo, the principle pattern used to describe a patient’s presentation includes descriptors from three modules: deficiency-excess, heat-cold, and optional body constituents (Yakubo et al., 2014). Deficiency-excess and heat-cold are essential and typically sufficient to describe most patient disease states. However, body constituents modules can be added for complicated health conditions and chronic diseases (detailed explanation in Supplementary Figure S1).

Pattern diagnosis and disease diagnosis are made simultaneously in Kampo clinic. Usually, a patient who visits a modern Kampo clinic will have one or more CM diagnoses when referred. After a Kampo-specific history and physical examination a pattern diagnosis will be given. The treating Kampo physician can also add several additional CM diagnoses if specific unique Kampo symptoms or physical exam signs are not covered within a patient’s previous CM diagnoses. Thus, patients who visit a Kampo clinic in Japan can end up with CM diseases diagnoses and a pattern diagnosis together. This would include one or more CM diseases from the ICD-10 and one Kampo pattern composed of one descriptor from deficiency-excess, one from heat-cold, and one or more optional descriptors from body constituents.

It has not been possible to compare standardized codes between TM and CM before the advent of the ICD-11. Historically, TM and CM are completely different medical systems, and TM considers the holistic state of a patient presentation rather than specific organ systems in CM. Furthermore, TM and CM have differing theoretical foundations, which suggests that there is no correlative overlap between the two systems. When TM pattern diagnoses are given, they do not include elements of a CM diagnosis.

However, modern-day Kampo clinical practice has evolved in a way that utilizes some knowledge of CM, and especially since physicians who specialize in Kampo medicine are required to be licensed CM practitioners. Thus, we hypothesized there were potential undiscovered relationships between the two medical systems. To this end, we conducted an exploratory cross-sectional study at the Kampo clinic of Keio University Hospital to understand and compare the characteristics and relationships between CM ICD-10 chapters and TM pattern descriptors.

## Methods

### Study Design

This is an exploratory cross-sectional study conducted at Keio University Hospital in Tokyo, Japan. Keio University Hospital is one of the largest and well-known teaching hospitals in Tokyo and houses a large and active Kampo clinic, making it an ideal location to obtain a wide range of different patient presentations. The Keio University School of Medicine Institutional Review Board approved this study (Approval No. 20100144), and the protocol is available at the UMIN clinical trials registry (unique ID: UMIN000020478).

### Participants

To avoid information bias from repeatedly collecting diagnostic information in the medical record, we set our inclusion criteria as first-visit patients aged 20 or older presenting to the Kampo clinic at Keio University Hospital from October 1st, 2014 to June 30th, 2019. Exclusion criteria included records without CM diagnoses or incomplete documentation of deficiency-excess or heat-cold. Both deficiency-excess and heat-cold modules are essential in the practice of Kampo medicine. There were 456 (37.7%) patients with a single CM diagnosis, 428 (35.4%) with two, and 325 (26.9%) with more than three in our database (detailed diseases and ICD-10 chapters distribution in Supplementary Tables S1,S2). To reflect the real-world distribution of pattern diagnose and obtain sufficient statistical power, we decided to include data from patients with multiple CM diagnoses. Written informed consent was obtained from participants. There was a total of 10 physicians included in this study, who were the practicing Kampo physicians at our institution. All the participating physicians are board-certified specialists in Kampo medicine who also have active conventional medicine licenses. Inter-rater reliability of some of these physicians has been shown previously (Maeda-Minami et al., 2021).

### Procedure

Before seeing a Kampo physician, all patients completed a standardized questionnaire, which assessed subjective symptoms and collected demographic information at their first visit. Based on the questionnaire, one Kampo physician would ask additional clarifying questions and conduct a series of Kampo physical examinations, such as tongue inspection, pulse diagnosis, and abdominal palpation. The physicians would then give their pattern diagnosis with three modules: deficiency-excess, heat-cold, and optional body constituents. In Kampo medicine, within the TM ICD-11 pattern chapter, the deficiency-excess module included deficiency, medium, and excess descriptors; the heat-cold module included heat, tangled, moderate, and cold descriptors; and the body constituents module included qi deficiency, qi stagnation, qi counterflow, blood deficiency, blood stasis, fluid disturbance, fluid deficiency, and kidney qi deficiency descriptors (Supplementary Figure S1). This pattern diagnosis was given without regard to the CM diagnosis and was made independently using the TM evaluation. We did consider pattern diagnosis interrater reliability when considering our study’s wider internal validity, however a study done previously by our group indicated this was not a major concern (Maeda-Minami et al., 2021).

CM diagnoses for patients visiting the Kampo clinic were coded using the ICD-10, released in 2013 by the Ministry of Health, Labor and Welfare in Japan (Ministry of Health Labor and Welfare., 2020). The number of conventional medicine diagnoses in ICD-10 is over 14,000, with no classifications for disease severity. Due to the difficulty in analyzing all >14,000 diseases in our analysis, CM diagnostic codes were grouped by ICD-10 chapters.

Information abstracted for analysis includes answers from the patient’s questionnaire, findings from Kampo physical examination, and the final pattern diagnosis. Chart review was also conducted to confirm the CM diagnosis and to record demographic characteristics such as age and gender.

### Statistical Analysis

All statistical calculations and analyses were performed using R software (version 3.6.3, 2020-02-29). Descriptive statistics were used in the patient’s demographic characteristics. The proportion of pattern code distribution by each CM ICD-10 chapter was illustrated by a bar plot. A test for equal proportions with continuity correction was used to compare the participant number distribution and sex distribution characteristics. Age and body mass index (BMI) characteristics were summarized as an interquartile range (IQR). Wilcoxon’s rank-sum test with continuity correction was used to compare the age and BMI characteristics due to non-normal distributions, comparing groups with a specific code diagnosis against groups without that code diagnosis. A *p-*value < 0.05 as statistically significant.

## Results

There were 1,568 potential study patients identified at the Keio University Hospital Kampo clinic between October 1st, 2014, and June 30th, 2019 (Supplementary Figure S2). Out of those 1,568, 1,319 patients (84.1%) agreed to participate in the study. There were 110 patients (8.3%) excluded due to incomplete diagnosis information, with 24 patients without deficiency-excess or heat-cold module, 12 without CM diagnoses, and 74 without both. There were 1,209 patients included in our final analysis.

### Characteristics of Kampo Clinic Patients

Sex ratio of the 1,209 patients was 1:2.7 (327 males: 882 females). Age of patients ranged from 20 to 92, and BMI from 12.8 to 52.5 kg/m^2^.

We report the distribution by each CM ICD-10 chapter (Supplementary Table S3; with top three most frequent CM diagnoses). The most common chapter was XVIII (*n* = 474, 39.2%), for symptoms, signs, and abnormal clinical and laboratory findings. CM ICD-10 chapters showed different distribution dependent on sex, age, and BMI. The proportion of males was higher in diseases of the circulatory system (IX, 40.7 vs 25.6%, and *p* < 0.01), and lower in diseases of the genitourinary system (XIV, 13.2 vs 30.54%, and *p* < 0.01). Age was more likely to be older in chapter IX (median 67 vs 51, *p* < 0.01), and more likely to be younger in chapter XII (diseases of the skin and subcutaneous tissue, median 41 vs 54, and *p* < 0.01). BMI was also more likely to be larger in chapter IX (median 23.1 vs 20.8, *p* < 0.01).

For pattern diagnoses (Supplementary Table S4), the most common descriptor within the deficiency-excess module was deficiency (*n* = 485, 40.1%). Within heat-cold, cold was the most common (*n* = 489, 40.4%). Within body constituents, qi stagnation was the most common (*n* = 369, 30.5%).

Pattern descriptor had different distributions depending on sex, age, and BMI (Supplementary Table S4). Within excess-deficiency, deficiency was more likely to be coded in elderly patients (median 57 vs 50, *p* < 0.01) and smaller BMI (median 19.2 vs 22.1, *p* < 0.01). Excess was greater in males (37.9 vs 24.1%, *p* < 0.01) and larger BMI (median 24 vs 20.2, *p* < 0.01).

Within heat-cold, cold was less likely to be in male patients (22.5 vs 30.1%, *p* < 0.01). Furthermore, patients with larger BMI were more likely to be given a heat descriptor (median 23.9 vs 20.8, *p* < 0.01) and younger patients given a tangled descriptor (median 49 vs 53, *p* < 0.01).

Within body constituents, qi deficiency was more likely to be with smaller BMI (median 19.6 vs 21.3, *p* < 0.01). Qi stagnation was more likely to be in younger patients (median 46 vs 55, *p* < 0.01). Blood stasis was more likely to be in younger patients (median 45 vs 55, *p* < 0.01) and had fewer male patients (12.2 vs 31.8%, *p* < 0.01). Fluid disturbance was also less likely in males (11.0 vs 30.1%, *p* < 0.01). Kidney qi deficiency tended to be in older (median 70 vs 48, *p* < 0.01) and male patients (50.2 vs 21.2%, *p* < 0.01).

### Relationship Between CM ICD-10 Chapters and TM ICD-11 Pattern Codes

**FIGURE 1:**
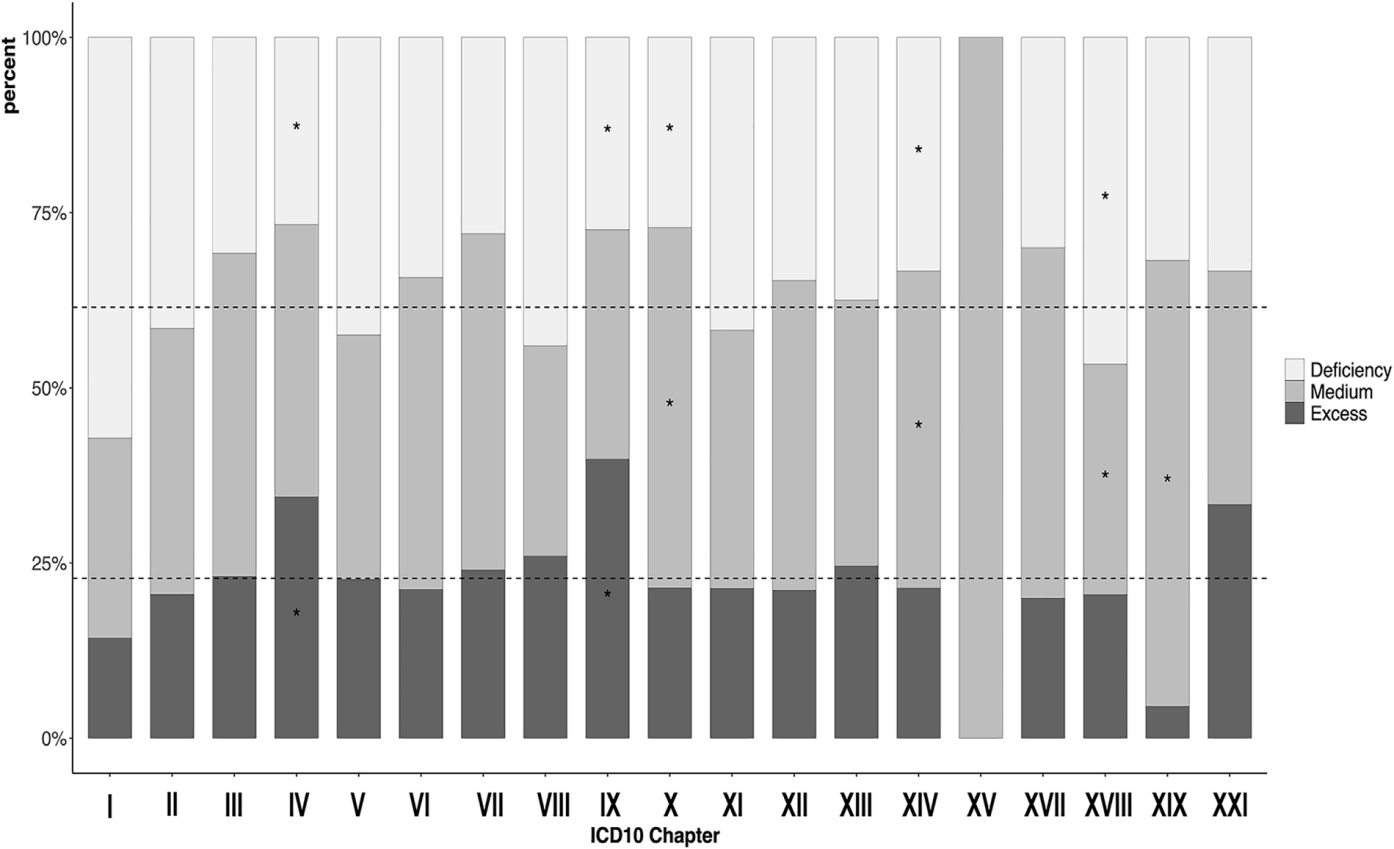
Proportion of participants with deficiency-excess in each the 10th version of the International Classification of Diseases chapters. *p < 0.05 ICD = International Classification of Diseases. I: Infection (35); II: Neoplasm (200); III: Hemotology/Immunology (13); IV: Endocrine/metabolism (90); V: Psychiatry (106); VI: Neurology (146); VII: Ophthalmology (25); VIII:Audiology (50); IX: Circulatology (113); X: Pulmonology (70); XI: Gastroenterology (206); XII: Dermatology (147); XIII: Musculoskeletal (224); XIV: Genitourology (243); XV: Obstetrics (3); XVII: Congenital (10); XVIII: Symptoms/signs (474); XIX: Injury/poisoning (22); XXI: Health state (3).

**FIGURE 2:**
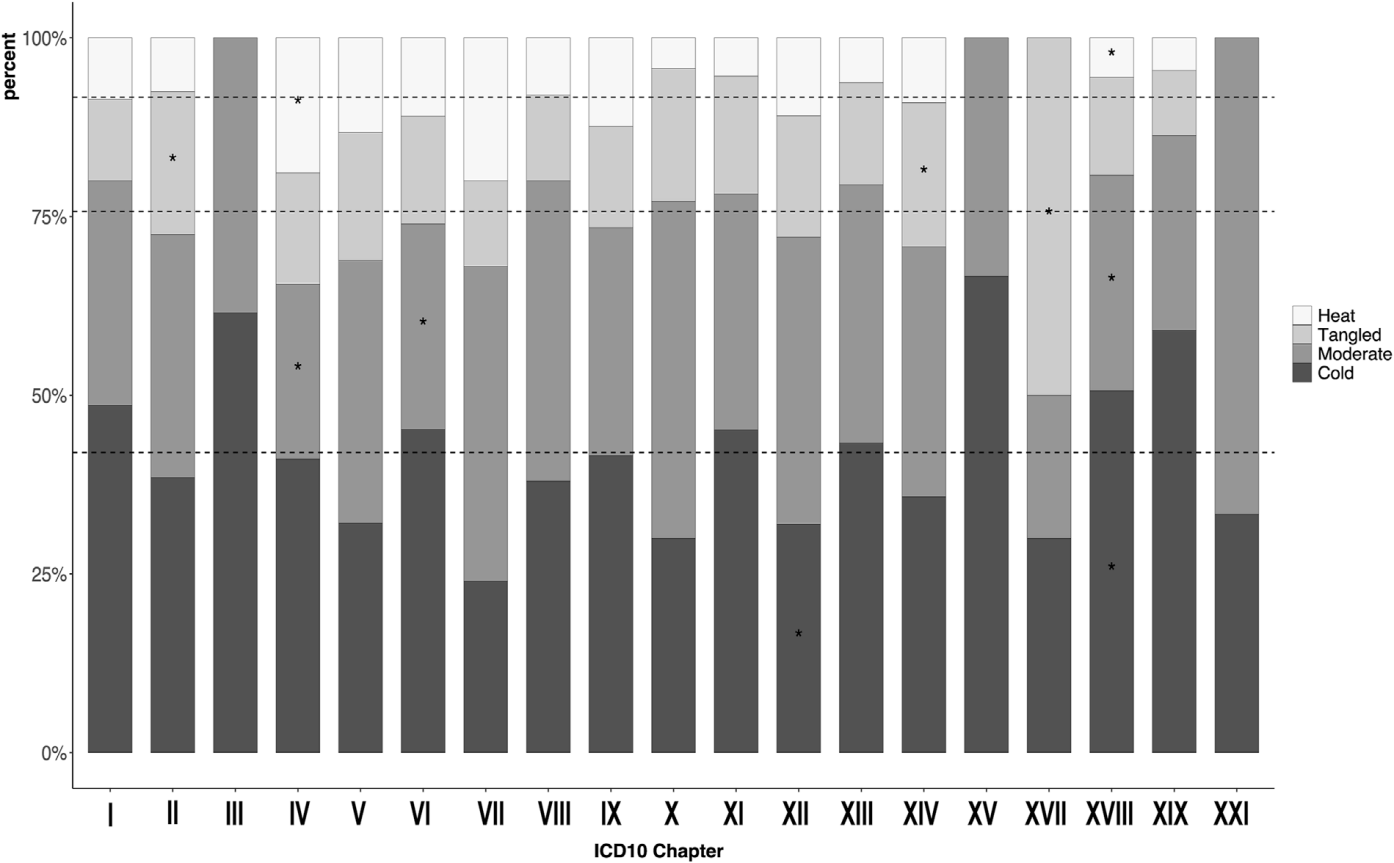
Proportion of participants with heat-cold pattern in each the 10th version of the International Classification of Diseases chapters. *p < 0.05 ICD = International Classification of Diseases. I: Infection (35); II: Neoplasm (200); III: Hemotology/Immunology (13); IV: Endocrine/metabolism (90); V: Psychiatry (106); VI: Neurology (146); VII: Ophthalmology (25); VIII:Audiology (50); IX: Circulatology (113); X: Pulmonology (70); XI: Gastroenterology (206); XII: Dermatology (147); XIII: Musculoskeletal (224); XIV: Genitourology (243); XV: Obstetrics (3); XVII: Congenital (10); XVIII: Symptoms/signs (474); XIX: Injury/poisoning (22); XXI: Health state (3).

**FIGURE 3:**
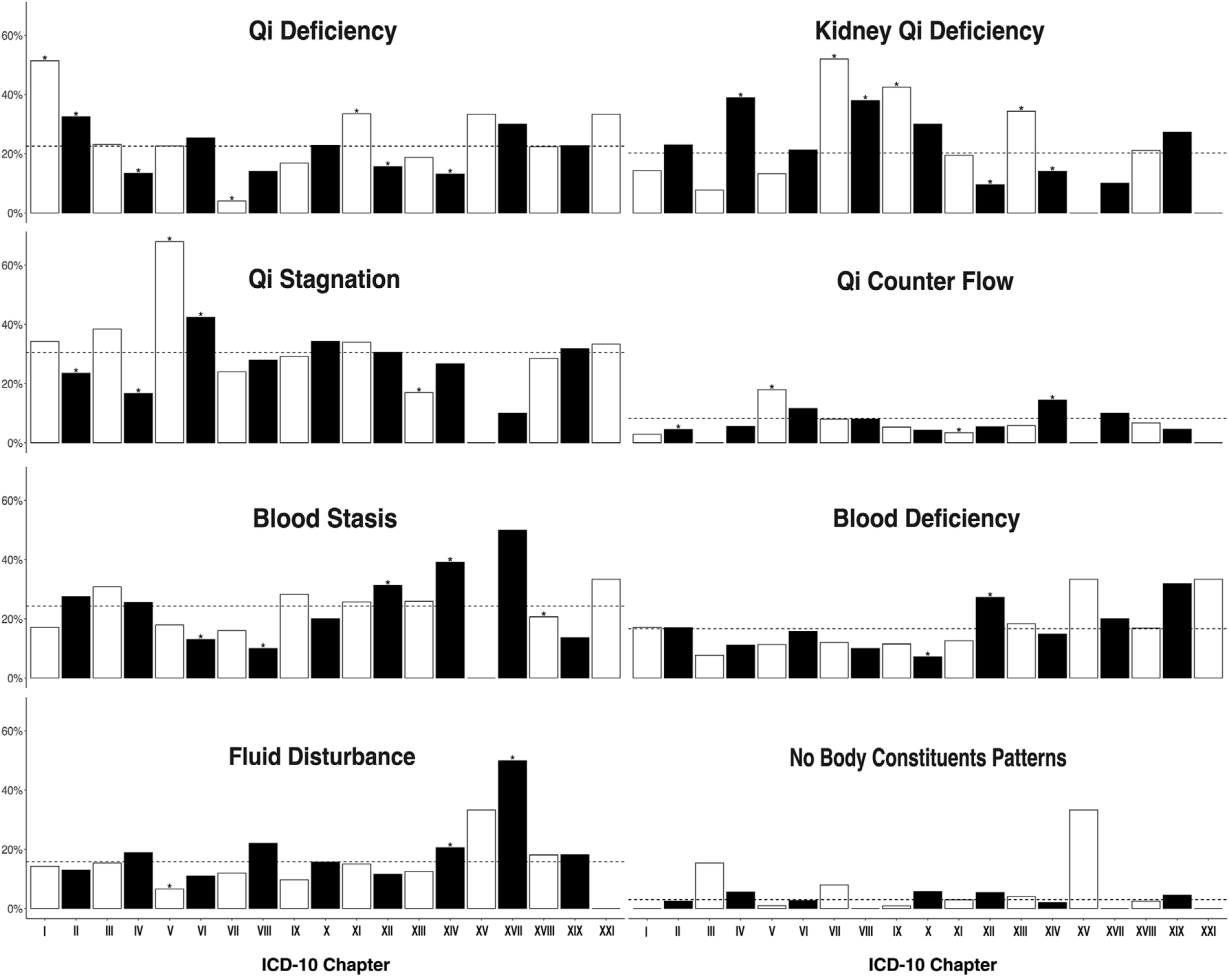
Proportion of participants with body constituents pattern in each the 10th version of the International Classification of Diseases chapters. *p < 0.05 ICD = International Classification of Diseases. The facet of fluid deficiency is hidden due to its limited participant number (*n* = 4). I: Infection (35); II: Neoplasm (200); III: Hemotology/Immunology (13); IV: Endocrine/metabolism (90); V: Psychiatry (106); VI: Neurology (146); VII: Ophthalmology (25); VIII:Audiology (50); IX: Circulatology (113); X: Pulmonology (70); XI: Gastroenterology (206); XII: Dermatology (147); XIII: Musculoskeletal (224); XIV: Genitourology (243); XV: Obstetrics (3); XVII: Congenital (10); XVIII: Symptoms/signs (474); XIX: Injury/poisoning (22); XXI: Health state (3).

In general, while each CM ICD-10 chapter (minus those that are not seen commonly in Kampo practice, such as chapter XX External causes of morbidity) had a general distribution of all deficiency-excess (Figure 1), heat-cold (Figure 2), and body constituents descriptors (Figure 3), our analysis found certain overrepresentations of specific Kampo pattern codes within each chapter.

Figure 1 shows the proportion of the deficiency-excess by each CM ICD-10 chapter, where scattered lines represent the average value in each descriptor. The proportion of deficiency in chapter XVIII (Symptoms, signs and abnormal clinical and laboratory findings, and not elsewhere classified) exceeded the other chapters (46.6 vs 35.9%, *p* < 0.01), in which cold hypersensitivity, headache, dizziness, and giddiness represent the majority of CM ICD-10 codes (Supplemental Table S3). The proportion of excess in chapter IV (Endocrine, nutritional, and metabolic diseases) and chapter IX (Diseases of the circulatory system) exceeded the other chapters (34.4 vs 20.1%, *p* < 0.01 and 39.8 vs 19.3%, *p* < 0.01 respectively), in which diabetes and hyperlipidemia represent the majority of codes in chapter IV and hypertension in chapter IX.

Figure 2 shows the proportion of the heat-cold by each CM ICD-10 chapter. Compared to other chapters, chapter XVIII had an excess of cold (50.6 vs 33.9%, *p* < 0.01). Chapter IV had an excess of heat (18.9 vs 7.1%, *p* < 0.01).

We analyzed body constituents by CM ICD-10 chapter and stratified each descriptor (Figure 3). There were three chapters that were overrepresented with qi deficiency: chapter I (Certain infectious and parasitic diseases, 51.4 vs 21.7%, *p* < 0.01), chapter II (Neoplasms, 32.5 vs 20.6%, *p* < 0.01, and chapter XI (Diseases of the digestive system, 33.5 vs 20.3%, *p* < 0.01). Infectious gastroenteritis and colitis were the most common ICD-10 codes in chapter II. Malignant breast tumor, leiomyoma of the uterus, and gastric tumor were the most common codes in chapter II. Constipation, chronic gastritis, and gastroesophageal reflux disease with esophagitis were the most common codes in chapter XI.

There were five chapters that were overrepresented with kidney qi deficiency: chapter IV (38.8 vs 18.8%, *p* < 0.5), chapter IX (42.5 vs 18.0%, *p* < 0.01), chapter VII (Diseases of the eye and adnexa, 52 vs 19.6%, *p* < 0.01), chapter VIII (Diseases of the ear and mastoid process, 38 vs 19.5%, *p* < 0.01), and chapter XIII (Diseases of the musculoskeletal system and connective tissue, 34.4 vs 17.1%, *p* < 0.01). Glaucoma was the most common CM ICD-10 code in chapter VII, tinnitus the most common ICD-10 code in chapter VIII, and lower back pain, shoulder stiffness and sicca syndrome the most common in chapter XIII. Chapter XII (Diseases of the skin and subcutaneous tissue) and XIV (Diseases of the genitourinary system) were less associated with kidney qi deficiency (9.5 vs 21.8%, *p* < 0.01 and 14.0 vs 21.8% respectively, both *p* < 0.01). In chapter XIV (menopausal and female climacteric states), unspecified female infertility, and unspecified dysmenorrhea were the most common ICD-10 codes represented.

There were two chapters that were overrepresented with qi stagnation, chapter V (Mental and behavioral disorders, 67.9 vs 26.9%, *p* < 0.01), and chapter VI (Diseases of the nervous system, 42.5 vs 28.8%, *p* < 0.01). Depressive disorder and anxiety were the most common CM ICD-10 codes represented in chapter V, and insomnias, disorder of the autonomic nervous system, and polyneuropathy in chapter VI.

There were two chapters overrepresented with qi counter flow, chapter V (17.9 vs 7.3%, *p* < 0.01) and chapter XIV (14.4 vs 6.7%, *p* < 0.01). Chapter XIII was less likely to be associated with qi counter flow pattern (17.0 vs 33.6%, *p* < 0.01).

Chapter XIV diagnoses were more likely to be associated with blood stasis (39.1 vs 20.6%, *p* < 0.01). Chapter XII diagnoses were more likely to be associated with blood deficiency (27.2 vs 15.2%, *p* < 0.01). Chapter V diagnoses were less likely to be associated with fluid disturbance (6.6 vs 16.7%, *p* < 0.01).

## Discussion

This is the first study in the literature to systematically examine the relationship between CM ICD-10 diagnoses and TM pattern diagnoses using a standard classification scheme in real-world clinical practice. Our results show that all pattern descriptors were represented within the spectrum of CM ICD-10 chapters. We report certain specific overrepresentations of pattern descriptors within each ICD-10 chapter and when stratified by age, sex, and BMI.

We found diseases relating to the eye or ear (ICD-10 chapter VII or VIII) were more likely to be associated with kidney qi deficiency. This relationship follows the Kampo theory that kidney qi nourishes the eye and ear function at baseline, and an absence of kidney qi is seen in eye and ear pathology. Another trend noted was that the population of chapter XIV (Diseases of the genitourinary system) was composed of two different groups: older males in whom the prevalent disease was prostate hypertrophy, and younger females in whom menopausal and female climacteric states were the primary diseases. The pattern descriptors distribution within chapter XIV also varied by sex which may indicate an association between these CM diagnoses and TM patterns—males were more likely to be associated with kidney qi deficiency, while females were more likely to be associated with blood stasis and fluid disturbance. We report the characteristics of patients with similar CM and TM diagnoses in Supplementary Tables S6–S8 and visually represent their differences in Supplementary Figures S3–S5.

Reference guide of ICD-11 articulates that ICD-11’s chapter on Traditional Medicine disorders and patterns is designed to be integrated with coding of cases in conjunction with the CM concepts of ICD Chapters. The relationship between TM pattern descriptors and CM diagnosis has been an established research topic within the East Asian TM community (Lu and Chen, 2011; Lu et al., 2012). Studies have shown the relationship of specific TM pattern descriptors with a diverse array of CM presentations, including knee osteoarthritis (Tian et al., 2016) and open-angle glaucoma (Yang et al., 2018). However, before the advent of the standardized TM ICD-11 pattern descriptors as in this study, it has been difficult to conduct a comparative analysis between differing medical traditions with unique terminology and classifications, and such as between Kampo and traditional Chinese medicine. These types of studies could be beneficial in-patient care. One study found that in patients with colorectal cancer, patients with deficiency descriptors had a higher survival rate than that of patients with excess descriptors—the authors hypothesized that patients diagnosed with excess could benefit from postoperative adjuvant chemotherapy (Li et al., 2020).

The standardized terminology in ICD-11 is a tool that allows for these cross-disciplinary comparisons to be made, both within TM in the different Asian modalities and between TM and CM. Regarding Kampo, while there exists much research into the efficacy of Kampo herbal formulas (Watanabe et al., 2011), much less is known about the significance between pattern diagnoses and their implications for CM diseases amongst TM patients. This will be a research field not only for Kampo practitioners, but for all TM practitioners in the future. The standardized terminology in the ICD-11 has already been utilized in other contexts by our group, such as in predictive modeling. Using the standardized TM pattern descriptors, we made a prediction model for cold-heat and deficiency-excess using data from a nation-wide sample of Kampo patients, and with the end goal to assist non-Kampo practitioners in making these Kampo diagnoses (Maeda-Minami et al., 2019; Maeda-Minami et al., 2020).

This study has some limitations. One limitation is its single-institutional design, reducing generalizability to the broader practice of Kampo in Japan and the world. It was also difficult to conduct stratified analyses for specific CM diagnoses due to our limited sample size. One future area of further research would be comparing TM with different CM disease severities–however there are no classifications for the disease severity in the ICD-10. In the ICD-11, severity of disease will be able to be classified using extension codes. Furthermore, once the ICD-11 is officially launched in 2022, and future research could utilize larger datasets from multiple countries for similar studies.

Our study was cross-sectional, not allowing us to capture how TM patterns may change over time. Also, mild diseases with short duration, such as the common cold or acute gastroenteritis, are not commonly seen in our clinic due to its affiliation in a university hospital—in our clinic, it is more common to see complex, and chronic disease states. We did not include adolescent and infant patients, limiting generalizability to the population below age 20. Finally, in this study, we only focused on the TM pattern descriptor relationship with CM chapters in ICD-10. Considering the holistic nature of TM and Kampo, future studies may seek to investigate the interactive relationships between different combinations of the three modules of Kampo patterns and CM diseases (Hu et al., 2011).

## Conclusion

We report Kampo pattern descriptors characteristics in TM ICD-11 pattern descriptors and compare these with CM chapters in ICD-10. Our findings show that certain Kampo pattern descriptors are related to specific CM ICD-10 chapters, especially after stratified by age, sex, and BMI. In the future, we hope that the results from this exploratory study motivate further investigation into the relationships between CM and TM diagnoses. This will be of importance to understand the relationship not only between Kampo and other TM modalities, but also the relationship between CM and TM, and how CM and TM can together advance patient care.

## Data Availability

The raw data supporting the conclusion of this article will be made available by the authors on request.
