# Supplementary material for: Relationship Between Conventional Medicine Chapters in ICD-10 and Kampo Pattern Diagnosis: A Cross-Sectional Study
Source: Front Pharmacol. 2021 Dec 20;12:751403. doi: 10.3389/fphar.2021.751403 (PMC8721141; doi:10.3389/fphar.2021.751403)
Supplement: Supplementary file 1 [file DataSheet2.docx]

**Supplementary Table 1**  Participants' distribution by the number of the coexisting diseases

| Disease | Count | Percent |
| --- | --- | --- |
| 1 | 456 | 37.7 |
| 2 | 428 | 35.4 |
| 3 | 201 | 16.6 |
| 4 | 78 | 6.5 |
| 5 | 31 | 2.6 |
| 6 | 6 | 0.5 |
| 7 | 6 | 0.5 |
| 8 | 2 | 0.2 |
| 11 | 1 | 0.1 |

**Supplementary Table 2** Participants’ distribution by the number of the coexisting ICD-10 chapters

| ICD-10 Chapter | Count | Percent |
| --- | --- | --- |
| 1 | 560 | 46.3 |
| 2 | 419 | 34.7 |
| 3 | 152 | 12.6 |
| 4 | 65 | 5.4 |
| 5 | 12 | 1.0 |
| 6 | 1 | 0.1 |

ICD-10= The 10th version of the International Classification of Diseases

**Supplementary Table 3** Demographics and characteristics of conventional medicine diagnoses of participants using ICD-10 chapters

| ICD-10 | Contents | N=1209 ^a^  (Male %) | | Age (years)  Median (IQR) | | BMI (kg/m^2^)  Median (IQR) | |
| --- | --- | --- | --- | --- | --- | --- | --- |
| I | Certain infectious and parasitic diseases | 35 | (17.1) | 60 | (39.5-66.0) | 19.2^*^ | (17.45-22.5) |
| II | Neoplasms | 200 | (30.5) | 56^**^ | (46-68.25) | 21 | (18.88-23.1) |
| III | Diseases of the blood and blood-forming organs and certain disorders involving the immune mechanism | 13 | (38.5) | 48 | (39-55) | 20.4 | (19.6-22) |
| IV | Endocrine, nutritional, and metabolic diseases | 90 | (37.8^**^) | 63^**^ | (51-73) | 22.8^**^ | (19.62-24.95) |
| V | Mental and behavioral disorders | 106 | (25.5) | 45^**^ | (38-60) | 21.1 | (18.7-23.75) |
| VI | Diseases of the nervous system | 146 | (22.6) | 55.5 | (41-72.75) | 20.85 | (19.02-22.8) |
| VII | Diseases of the eye and adnexa | 25 | (40.0) | 60^**^ | (51-72) | 22.3 | (19.6-24.5) |
| VIII | Diseases of the ear and mastoid process | 50 | (34.0) | 57.5^*^ | (50-71) | 21.6 | (19.6-23.25) |
| IX | Diseases of the circulatory system | 113 | (40.7^**^) | 67^**^ | (56-74) | 23.1^**^ | (20.3-24.3) |
| X | Diseases of the respiratory system | 70 | (38.6^*^) | 56.5 | (44.25-68.75) | 21.5 | (19.52-22.8) |
| XI | Diseases of the digestive system | 206 | (28.2) | 52.5 | (39-68.75) | 20.4^*^ | (18.62-22.6) |
| XII | Diseases of the skin and subcutaneous tissue | 147 | (27.2) | 41^**^ | (30-55) | 20.8 | (19.5-22.9) |
| XIII | Diseases of the musculoskeletal system and connective tissue | 224 | (26.8) | 65^**^ | (51-73) | 21.5 | (19.1-23.52) |
| XIV | Diseases of the genitourinary system | 243 | (13.2^**^) | 43^**^ | (37-53) | 20.7 | (18.7-22.9) |
| XV | Pregnancy, childbirth, and the puerperium | 3 | (0) | 43 | (39-55.5) | 20 | (19.85-20.9) |
| XVII | Congenital malformations, deformations, and chromosomal abnormalities | 10 | (20.0) | 49 | (33.75-61) | 21.35 | (20.4-22.83) |
| XVIII | Symptoms, signs, and abnormal clinical and laboratory findings, not elsewhere classified | 474 | (25.7^*^) | 55^**^ | (42-69) | 20.65^*^ | (18.7-23.1) |
| XIX | Injury, poisoning, and certain other consequences of external causes | 22 | (18.2) | 46 | (38.25-68.75) | 19.6 | (18.72-21.9) |
| XXI | Factors influencing health status and contact with health services | 3 | (33.3) | 35 | (35-45.5) | 20 | (19.9-22.15) |
| Total |  | 1209 | (27) | 52 | (40-67) | 21 | (19-23.2) |

^a^ Participants in these chapters are represented in more than one chapter.

ICD-10= The 10th version of the International Classification of Diseases

* *p* < 0.05, ** *p* < 0.01, compared within each pattern group.

**Supplementary Table 4** Demographics and characteristics of Kampo pattern descriptors assigned from ICD-11

| ICD-11 | N=1209 ^a^  (Male%) | | Age (years)  Median (IQR) | | BMI (kg/m^2^)  Median (IQR) | |
| --- | --- | --- | --- | --- | --- | --- |
| Deficiency | 485 | (22.1^**^) | 57^**^ | (20-92) | 19.2^**^ | (17.7-21.2) |
| Medium | 468 | (26.3) | 50^**^ | (20-91) | 21.2^**^ | (19.7-22.9) |
| Excess | 256 | (37.9^**^) | 50.5 | (20-90) | 23.8^**^ | (22-25.6) |
| Cold | 489 | (22.5^**^) | 56^*^ | (20-91) | 20^**^ | (18.4-22) |
| Moderate | 443 | (33.0^**^) | 52 | (20-90) | 21.5^**^ | (19.35-23.5) |
| Heat | 96 | (34.4) | 51 | (21-85) | 23.9^**^ | (21.35-26.3) |
| Tangled | 181 | (21.0) | 49^**^ | (22-92) | 21.4 | (19.6-23.1) |
| No body patterns | 37 | (40.5) | 51 | (21-82) | 21.8 | (20.1-23.5) |
| Qi Deficiency | 273 | (30.8) | 56^*^ | (21-89) | 19.6^**^ | (17.8-21.8) |
| Qi Stagnation | 369 | (29.3) | 46^**^ | (20-87) | 20.7 | (19.1-23.2) |
| Qi Counter Flow | 100 | (17.0^*^) | 50 | (21-92) | 20.4 | (18.7-22.42) |
| Blood Deficiency | 201 | (20.0^*^) | 51 | (20-91) | 20.4^**^ | (18.7-22.2) |
| Blood Stasis | 294 | (12.2^**^) | 45^**^ | (20-92) | 21.95^**^ | (19.9-24) |
| Fluid Disturbance | 191 | (11.0^**^) | 45^**^ | (21-88) | 21 | (18.95-23.25) |
| Fluid Deficiency | 4 | (25.0) | 63.5 | (40-84) | 21.75 | (19.52-24.48) |
| Kidney Qi Deficiency | 245 | (50.2^**^) | 70^**^ | (22-91) | 21.6^*^ | (19.5-23.4) |

^a^ Participants in these chapters are represented in more than one chapter.

* *p* < 0.05, ** *p* < 0.01, compared within each pattern group.

ICD-11= The 11th version of the International Classification of Diseases

Supplementary Table 5: Top 3 diseases in each ICD-10 chapter

| Chapter | Code | English name | Participant No |
| --- | --- | --- | --- |
| I | A09 | Infectious gastroenteritis and colitis, unspecified | 12 |
| I | A31.9 | Mycobacterial infection, unspecified | 9 |
| I | B02.2 | Zoster with other nervous system involvement | 4 |
| II | C50.9 | Malignant neoplasm: Breast, unspecified | 34 |
| II | D25.9 | Leiomyoma of uterus, unspecified | 29 |
| II | C16.9 | Malignant neoplasm of stomach, unspecified | 11 |
| III | D64.9 | Anemia, unspecified | 5 |
| III | D61.9 | Aplastic anemia, unspecified | 2 |
| III | D69.3 | Immune thrombocytopenic purpura | 2 |
| IV | E14 | Unspecified diabetes mellitus | 27 |
| IV | E78.5 | Hyperlipidemia, unspecified | 27 |
| IV | E06.3 | Autoimmune thyroiditis | 7 |
| V | F32.9 | Major depressive disorder, single episode, unspecified | 31 |
| V | F41.1 | Generalized anxiety disorder | 16 |
| V | F20.9 | Schizophrenia, unspecified | 7 |
| VI | G47.0 | Disorders of initiating and maintaining sleep [insomnias] | 68 |
| VI | G90.9 | Disorder of the autonomic nervous system, unspecified | 14 |
| VI | G62.9 | Polyneuropathy, unspecified | 12 |
| VII | H40.9 | Unspecified glaucoma | 10 |
| VII | H04.1 | Disorders of lacrimal gland | 3 |
| VII | H43.8 | Disorders of vitreous body | 2 |
| VIII | H93.1 | Tinnitus | 35 |
| VIII | H91.2 | Sudden idiopathic hearing loss | 7 |
| VIII | H91.9 | Hearing loss, unspecified | 6 |
| IX | I10 | Essential (primary) hypertension | 73 |
| IX | I20.9 | Angina pectoris, unspecified | 5 |
| IX | I83.9 | Varicose veins of lower extremities without ulcer or inflammation | 5 |
| X | J45.9 | Asthma, unspecified | 19 |
| X | J30.1 | Allergic rhinitis due to pollen | 12 |
| X | J30.4 | Allergic rhinitis, unspecified | 10 |
| XI | K59.0 | Constipation | 50 |
| XI | K29.5 | Chronic gastritis, unspecified | 30 |
| XI | K21.0 | Gastro-esophageal reflux disease with esophagitis | 19 |
| XII | L20.9 | Atopic dermatitis, unspecified | 32 |
| XII | L50.9 | Urticaria, unspecified | 23 |
| XII | L30.9 | Dermatitis, unspecified | 13 |
| XIII | M54.5 | Low back pain | 42 |
| XIII^*^ | M62.8 | Muscle weakness (generalized) (shoulder stiffness) | 19 |
| XIII | M35.0 | Sicca syndrome [Sjögren] | 14 |
| XIV | N95.1 | Menopausal and female climacteric states | 61 |
| XIV | N97.9 | Female infertility, unspecified | 41 |
| XIV | N94.6 | Dysmenorrhea, unspecified | 37 |
| XV | O21.0 | Mild hyperemesis gravidarum | 1 |
| XV | O82.9 | Delivery by caesarean section, unspecified | 1 |
| XV | O99.0 | Anaemia complicating pregnancy, childbirth and the puerperium | 1 |
| XVII | Q87.2 | Congenital malformation syndromes predominantly involving limbs | 3 |
| XVII | Q26.8 | Congenital malformations of great veins | 2 |
| XVII | Q21.1 | Atrial septal defect | 1 |
| XVIII^*^ | R68.8 | Other specified general symptoms and signs (Cold hypersensitivity) | 134 |
| XVIII | R51 | Headache | 44 |
| XVIII | R42 | Dizziness and giddiness | 36 |
| XIX^*^ | T69.1 | Chilblains (frostbite) | 4 |
| XIX | T08 | Fracture of spine, level unspecified | 2 |
| XIX | T14.2 | Fracture of unspecified body region | 2 |
| XXI | Z33 | Pregnant state | 2 |
| XXI* | Z90.4 | Acquired absence of other parts of digestive tract (post cholecystectomy) | 1 |

ICD-10 chapters and codes are based on the 2013 Japanese version from the Ministry of Health, Labour and Welfare (https://www.mhlw.go.jp/toukei/sippei/). English translation is based on the WHO ICD-10 browser (https://www.who.int/classifications/icd/icdonlineversions/en/).

*specific diseases used in Japan or Kampo clinic, translation is added behind the English version in parentheses.

Supplementary Table 6: Number of participants with similar CM and TM diagnoses

| Pattern descriptors | I | II | III | IV | V | VI | VII | VIII | IX | X | XI | XII | XIII | XIV | XV | XVII | XVIII | XIX | XXI |
| --- | --- | --- | --- | --- | --- | --- | --- | --- | --- | --- | --- | --- | --- | --- | --- | --- | --- | --- | --- |
| Deficiency | 20 | 83 | 4 | 24 | 45 | 50 | 7 | 22 | 31 | 19 | 86 | 51 | 84 | 81 | 0 | 3 | 221 | 7 | 1 |
| Medium | 10 | 76 | 6 | 35 | 37 | 65 | 12 | 15 | 37 | 36 | 76 | 65 | 85 | 110 | 3 | 5 | 156 | 14 | 1 |
| Excess | 5 | 41 | 3 | 31 | 24 | 31 | 6 | 13 | 45 | 15 | 44 | 31 | 55 | 52 | 0 | 2 | 97 | 1 | 1 |
| Cold | 17 | 77 | 8 | 37 | 34 | 66 | 6 | 19 | 47 | 21 | 93 | 47 | 97 | 87 | 2 | 3 | 240 | 13 | 1 |
| Moderate | 11 | 68 | 5 | 22 | 39 | 42 | 11 | 21 | 36 | 33 | 68 | 59 | 81 | 85 | 1 | 2 | 143 | 6 | 2 |
| Heat | 3 | 15 | 0 | 17 | 14 | 16 | 5 | 4 | 14 | 3 | 11 | 16 | 14 | 22 | 0 | 0 | 26 | 1 | 0 |
| Tangled | 4 | 40 | 0 | 14 | 19 | 22 | 3 | 6 | 16 | 13 | 34 | 25 | 32 | 49 | 0 | 5 | 65 | 2 | 0 |
| No body constituents patterns | 0 | 5 | 2 | 5 | 1 | 4 | 2 | 0 | 1 | 4 | 6 | 8 | 9 | 5 | 1 | 0 | 12 | 1 | 0 |
| Qi Deficiency | 18 | 65 | 3 | 12 | 24 | 37 | 1 | 7 | 19 | 16 | 69 | 23 | 42 | 32 | 1 | 3 | 106 | 5 | 1 |
| Qi Stagnation | 12 | 47 | 5 | 15 | 72 | 62 | 6 | 14 | 33 | 24 | 70 | 45 | 38 | 65 | 0 | 1 | 135 | 7 | 1 |
| Qi Counter Flow | 1 | 9 | 0 | 5 | 19 | 17 | 2 | 4 | 6 | 3 | 7 | 8 | 13 | 35 | 0 | 1 | 32 | 1 | 0 |
| Blood Deficiency | 6 | 34 | 1 | 10 | 12 | 23 | 3 | 5 | 13 | 5 | 26 | 40 | 41 | 36 | 1 | 2 | 80 | 7 | 1 |
| Blood Stasis | 6 | 55 | 4 | 23 | 19 | 19 | 4 | 5 | 32 | 14 | 53 | 46 | 58 | 95 | 0 | 5 | 98 | 3 | 1 |
| Fluid Disturbance | 5 | 26 | 2 | 17 | 7 | 16 | 3 | 11 | 11 | 11 | 31 | 17 | 28 | 50 | 1 | 5 | 86 | 4 | 0 |
| Fluid Deficiency | 0 | 0 | 0 | 1 | 1 | 1 | 0 | 0 | 2 | 0 | 2 | 0 | 2 | 0 | 0 | 0 | 4 | 0 | 0 |
| Kidney Qi Deficiency | 5 | 46 | 1 | 35 | 14 | 31 | 13 | 19 | 48 | 21 | 40 | 14 | 77 | 34 | 0 | 1 | 100 | 6 | 0 |

Supplementary Table 7: Median age of participants with similar CM and TM diagnoses

| Pattern descriptors | I | II | III | IV | V | VI | VII | VIII | IX | X | XI | XII | XIII | XIV | XV | XVII | XVIII | XIX | XXI |
| --- | --- | --- | --- | --- | --- | --- | --- | --- | --- | --- | --- | --- | --- | --- | --- | --- | --- | --- | --- |
| Deficiency | 61.5 | 61 | 43.5 | 64 | 55 | 57.5 | 67 | 58.5 | 61 | 59 | 53.5 | 46 | 66 | 42 | NA | 53 | 61 | 67 | 35 |
| Medium | 61 | 52.5 | 53.5 | 63 | 44 | 57 | 58.5 | 55 | 69 | 59 | 51 | 39 | 60 | 43 | 43 | 36 | 51 | 43 | 35 |
| Excess | 49 | 52 | 44 | 62 | 43.5 | 50 | 52.5 | 56 | 66 | 51 | 54 | 42 | 62 | 42.5 | NA | 72.5 | 52 | 44 | 56 |
| Cold | 63 | 58 | 44.5 | 70 | 48.5 | 58 | 65.5 | 55 | 67 | 57 | 54 | 44 | 66 | 41 | 39 | 55 | 58 | 59 | 35 |
| Moderate | 60 | 59.5 | 55 | 62.5 | 45 | 56 | 67 | 58 | 66 | 65 | 49.5 | 40 | 65 | 43 | 68 | 51.5 | 54 | 53.5 | 45.5 |
| Heat | 49 | 55 | NA | 61 | 51.5 | 46.5 | 51 | 64 | 61.5 | 39 | 46 | 38.5 | 57.5 | 51.5 | NA | NA | 56 | 44 | NA |
| Tangled | 61 | 48.5 | NA | 57.5 | 42 | 43.5 | 52 | 63.5 | 71 | 52 | 55.5 | 37 | 51.5 | 43 | NA | 45 | 49 | 39.5 | NA |
| No body constituents patterns | NA | 60 | 58.5 | 51 | 51 | 57.5 | 49.5 | NA | 75 | 57 | 50 | 48 | 48 | 48 | 68 | NA | 53.5 | 34 | NA |
| Qi Deficiency | 58.5 | 63 | 27 | 72.5 | 56 | 60 | 71 | 51 | 63 | 59 | 56 | 39 | 63 | 44.5 | 35 | 36 | 57 | 67 | 35 |
| Qi Stagnation | 60 | 52 | 44 | 57 | 43.5 | 45 | 64.5 | 55 | 65 | 46 | 53 | 40 | 57 | 41 | NA | 23 | 48 | 37 | 56 |
| Qi Counter Flow | 69 | 52 | NA | 45 | 45 | 50 | 50.5 | 54 | 63.5 | 45 | 50 | 44.5 | 55 | 50 | NA | 45 | 51.5 | 69 | NA |
| Blood Deficiency | 60 | 51 | 48 | 68 | 44 | 57 | 59 | 48 | 65 | 63 | 50 | 42 | 59 | 42 | 43 | 65.5 | 54.5 | 48 | 35 |
| Blood Stasis | 64 | 47 | 44.5 | 51 | 40 | 41 | 53 | 57 | 56 | 41.5 | 46 | 39 | 55.5 | 39 | NA | 36 | 50.5 | 36 | 35 |
| Fluid Disturbance | 53 | 48 | 44.5 | 60 | 44 | 40.5 | 61 | 50 | 62 | 49 | 41 | 34 | 61.5 | 40 | 43 | 45 | 45 | 42 | NA |
| Fluid Deficiency | NA | NA | NA | 71 | 56 | 40 | NA | NA | 63.5 | NA | 63.5 | NA | 48 | NA | NA | NA | 63.5 | NA | NA |
| Kidney Qi Deficiency | 69 | 69 | 81 | 73 | 58.5 | 74 | 68 | 66 | 73 | 70 | 72 | 70 | 73 | 67 | NA | 82 | 72 | 77 | NA |

Supplementary Table 8: Proportion of male participants with similar CM and TM diagnoses

| Pattern descriptors | I | II | III | IV | V | VI | VII | VIII | IX | X | XI | XII | XIII | XIV | XV | XVII | XVIII | XIX | XXI |
| --- | --- | --- | --- | --- | --- | --- | --- | --- | --- | --- | --- | --- | --- | --- | --- | --- | --- | --- | --- |
| Deficiency | 10 | 33.73 | 25 | 25 | 17.78 | 14 | 28.57 | 9.09 | 22.58 | 31.58 | 23.26 | 21.57 | 16.67 | 7.41 | NA | 33.33 | 22.17 | 14.29 | 0 |
| Medium | 30 | 30.26 | 50 | 48.57 | 32.43 | 23.08 | 25 | 53.33 | 45.95 | 27.78 | 27.63 | 21.54 | 29.41 | 16.36 | 0 | 0 | 24.36 | 14.29 | 0 |
| Excess | 20 | 24.39 | 33.33 | 35.48 | 29.17 | 35.48 | 83.33 | 53.85 | 48.89 | 73.33 | 38.64 | 48.39 | 38.18 | 15.38 | NA | 50 | 36.08 | 100 | 100 |
| Cold | 5.88 | 31.17 | 37.5 | 40.54 | 14.71 | 22.73 | 50 | 15.79 | 38.3 | 33.33 | 20.43 | 10.64 | 25.77 | 13.79 | 0 | 0 | 23.33 | 15.38 | 0 |
| Moderate | 36.36 | 41.18 | 40 | 50 | 35.9 | 26.19 | 36.36 | 42.86 | 50 | 42.42 | 36.76 | 32.2 | 33.33 | 17.65 | 0 | 0 | 30.77 | 33.33 | 50 |
| Heat | 0 | 20 | NA | 35.29 | 42.86 | 25 | 40 | 75 | 28.57 | 66.67 | 36.36 | 50 | 42.86 | 13.64 | NA | NA | 30.77 | 0 | NA |
| Tangled | 25 | 15 | NA | 14.29 | 10.53 | 13.64 | 33.33 | 33.33 | 37.5 | 30.77 | 29.41 | 32 | 6.25 | 4.08 | NA | 40 | 21.54 | 0 | NA |
| No body constituents patterns | NA | 40 | 100 | 60 | 100 | 0 | 100 | NA | 0 | 25 | 33.33 | 50 | 22.22 | 40 | 0 | NA | 33.33 | 0 | NA |
| Qi Deficiency | 16.67 | 50.77 | 66.67 | 33.33 | 29.17 | 16.22 | 0 | 14.29 | 31.58 | 43.75 | 30.43 | 26.09 | 19.05 | 15.63 | 0 | 33.33 | 30.19 | 20 | 0 |
| Qi Stagnation | 16.67 | 21.28 | 40 | 33.33 | 25 | 24.19 | 50 | 28.57 | 48.48 | 37.5 | 32.86 | 35.56 | 23.68 | 6.15 | NA | 0 | 26.67 | 14.29 | 100 |
| Qi Counter Flow | 0 | 0 | NA | 20 | 21.05 | 23.53 | 50 | 25 | 16.67 | 0 | 14.29 | 12.5 | 15.38 | 8.57 | NA | 0 | 25 | 0 | NA |
| Blood Deficiency | 33.33 | 35.29 | 0 | 10 | 33.33 | 17.39 | 0 | 40 | 38.46 | 40 | 19.23 | 17.5 | 17.07 | 2.78 | 0 | 50 | 15 | 0 | 0 |
| Blood Stasis | 16.67 | 7.27 | 0 | 30.43 | 15.79 | 5.26 | 0 | 20 | 28.13 | 7.14 | 9.43 | 13.04 | 18.97 | 2.11 | NA | 20 | 11.22 | 0 | 0 |
| Fluid Disturbance | 20 | 0 | 0 | 17.65 | 0 | 6.25 | 0 | 45.45 | 27.27 | 18.18 | 12.9 | 17.65 | 10.71 | 0 | 0 | 20 | 10.47 | 0 | NA |
| Fluid Deficiency | NA | NA | NA | 100 | 0 | 0 | NA | NA | 50 | NA | 50 | NA | 0 | NA | NA | NA | 25 | NA | NA |
| Kidney Qi Deficiency | 0 | 52.17 | 0 | 62.86 | 42.86 | 45.16 | 53.85 | 47.37 | 52.08 | 61.9 | 47.5 | 42.86 | 45.45 | 76.47 | NA | 0 | 47 | 50 | NA |
