# Supplementary material for: Relationship Between Conventional Medicine Chapters in ICD-10 and Kampo Pattern Diagnosis: A Cross-Sectional Study
Source: Front Pharmacol. 2021 Dec 20;12:751403. doi: 10.3389/fphar.2021.751403 (PMC8721141; doi:10.3389/fphar.2021.751403)
Supplement: Supplementary file 2 [file DataSheet1.docx]

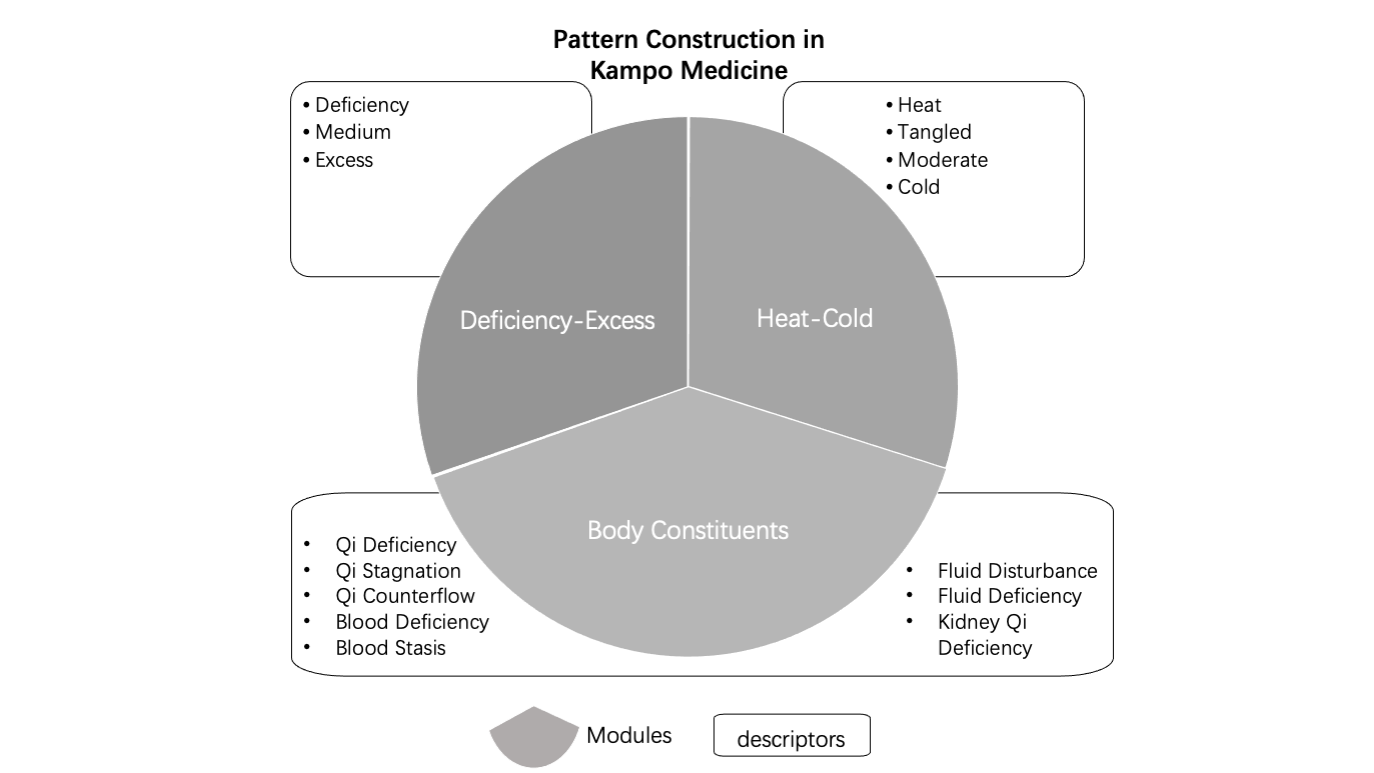


**Supplementary Figure 1** Pattern construction in Kampo medicine

Deficiency-Excess module is based on the patient’s body energy. Deficiency means low body energy, while excess means overactive energy status. Medium means neutral in “deficiency and excess”.

Heat-Cold module is based on the repairing responses condition. Heat means febrile status, while cold means chilly status. Moderate means neutral in “cold and heat”. Tangled is the mixture “cold and heat,” for example, cold feet and hot flush on face.

Body constituents module is to describe the Qi/ Blood/ Fluid/ Kidney Qi functions especially in chronic diseases. Qi deficiency means that Qi is not sufficient (strong) enough to sustain his/her living functions. Qi stagnation/ Qi counterflow means Qi abnormal movement; Qi stagnation is in depression, heavy headedness or nausea in Western terms; Qi counterflow is likely to be seen in patients with palpitations, irritation or paroxysmal headache. Blood deficiency means that blood is not sufficient, often seen in pallor, dizziness or alopecia in Western terms. Blood stasis refers to the blood stagnation, in such conditions as lower limb varix, telangiectasia and pigmentation of the skin. Fluid disturbance is the fluid stagnation, in such conditions as dizziness, edema and swelling of the tongue. Fluid deficiency is the lack of fluid. Kidney Qi deficiency is the lack of Kidney Qi.

Screened adult patients in appointment system

at Kampo clinic between 2014/10-2019/06

N = 1568

Exclusion due to incomplete data

N = 110

(24 incomplete Kampo pattern diagnoses,

12 incomplete CM diagnoses,

74 with both)

Patients analyzed

N = 1209

Registered patients

N = 1319

**Supplementary Figure 2** Participant recruitment flow chart

CM = conventional medicine.


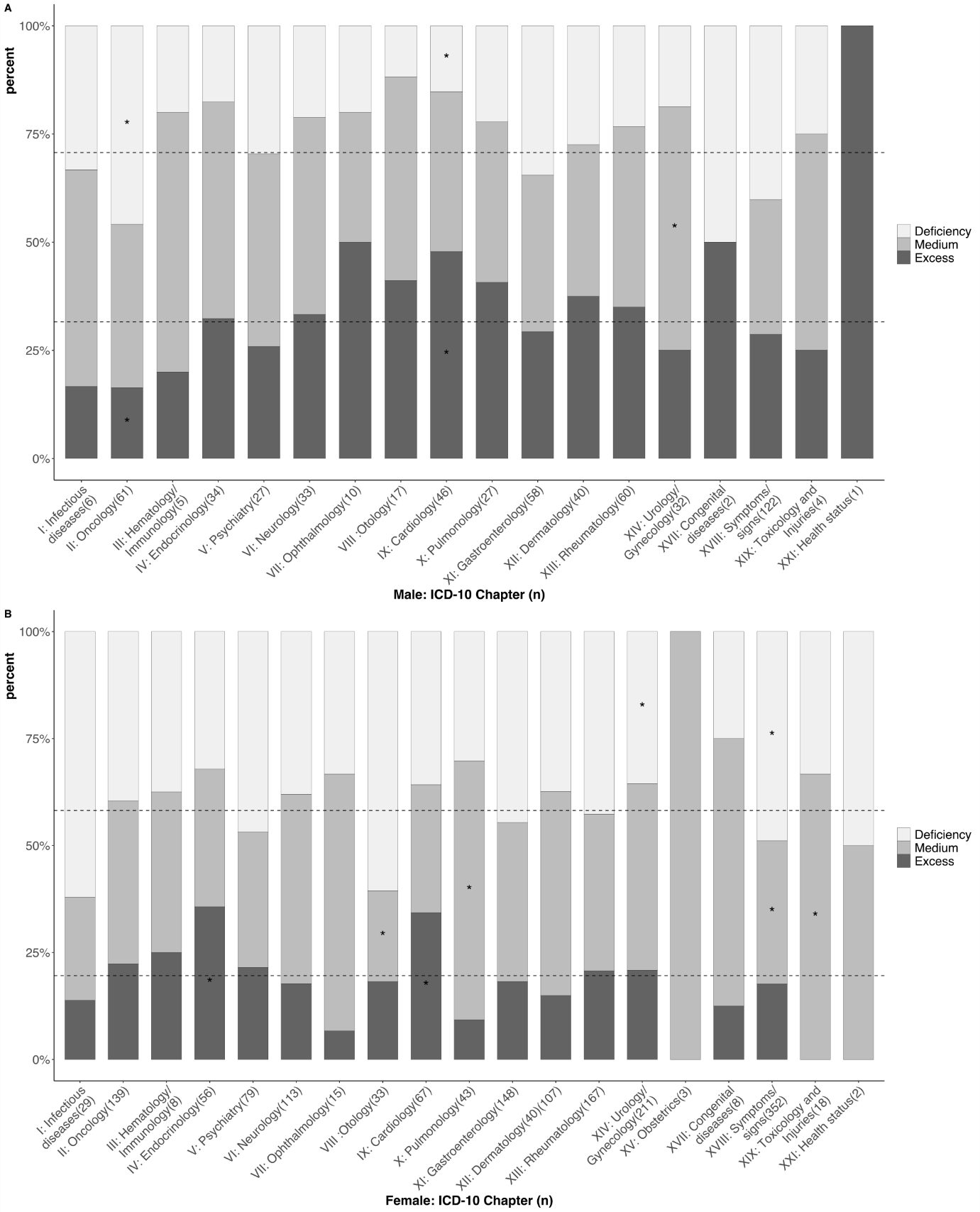


**Supplementary Figure 3** Proportion of deficiency-excess in male (A) and female (B) participants in each of the 10th Version of International Classification of Diseases chapters

* *p* < 0.05 ICD = International Classification of Diseases

Supplementary Figure 3 shows the proportion of each CM chapter's deficiency-excess in the ICD-10. The proportion of deficiency in chapter XVIII (Symptoms, signs and abnormal clinical and laboratory findings, not elsewhere classified), which exceeded the other chapters in Figure 1, was only seen in female groups. The proportion of excess in chapter IV (Endocrine, nutritional and metabolic diseases) was only significantly overrepresented in females, but in chapter IX (Diseases of the circulatory system) was overrepresented in both genders.


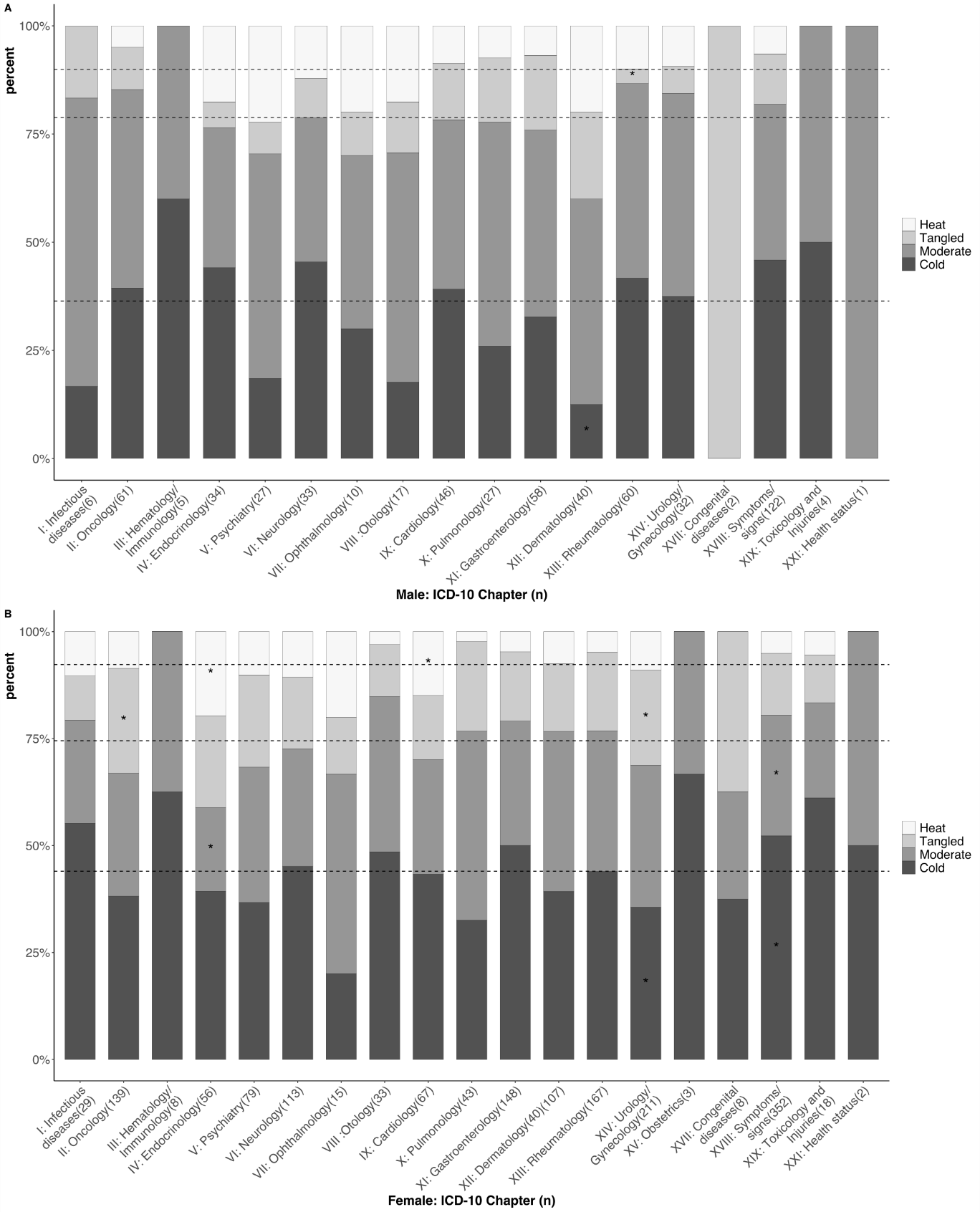


**Supplementary Figure 4** Proportion of heat-cold in male (A) and female (B) participants in each of the 10th version of International Classification of Diseases chapters

* *p* < 0.05 ICD = International Classification of Diseases

Supplementary Figure 4 shows the proportion of heat-cold. Chapter XVIII had a majority of cold in Figure 2, which was only significant in females. Chapter IV, which had a large heat proportion among both males and females, was not significantly greater among males potentially due to a small sample of males.


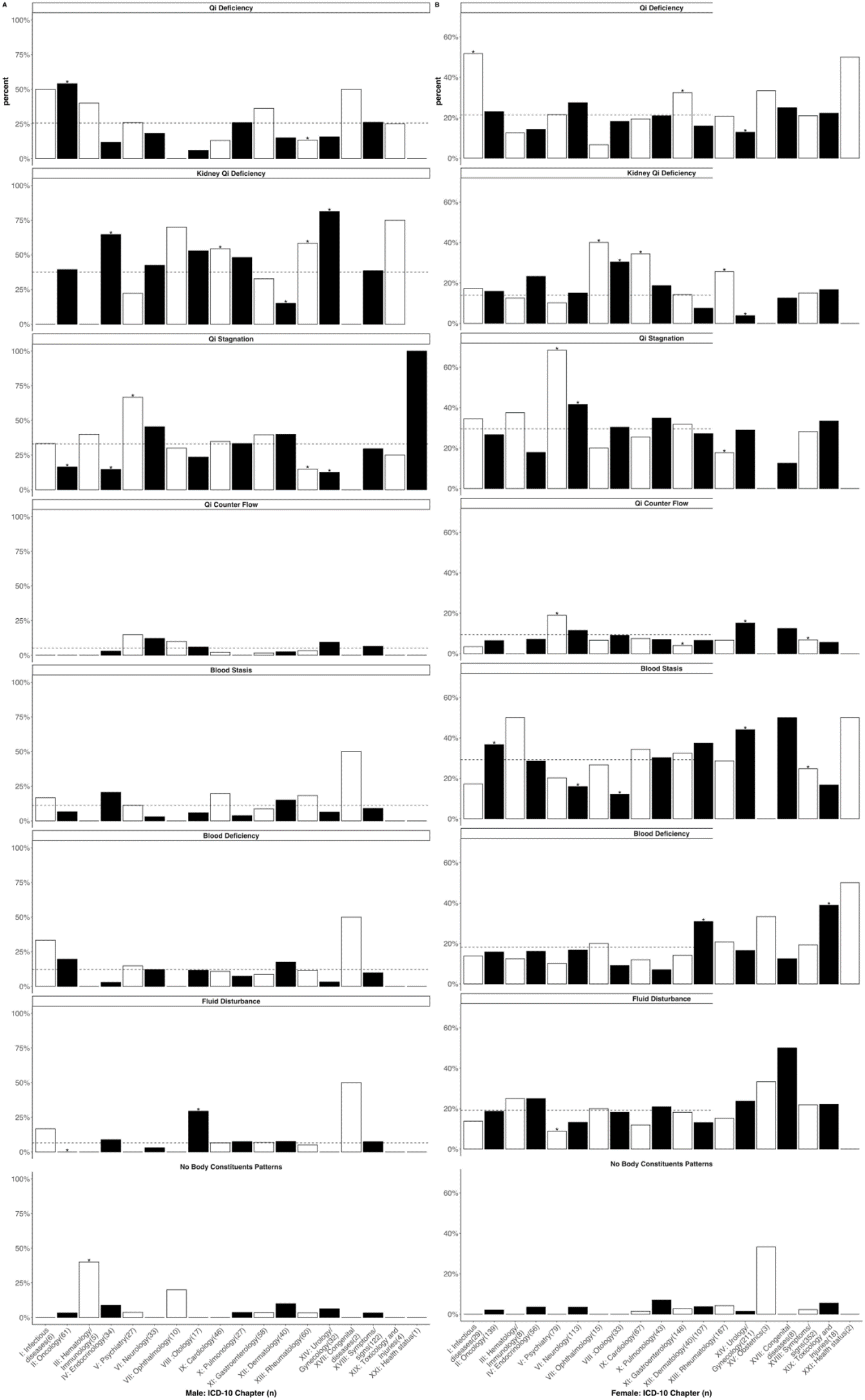


**Supplementary Figure 5** Proportion of body constituents in male (A) and female (B) participants in each of the 10th Version of International Classification of Diseases chapters

* *p* < 0.05 ICD = International Classification of Diseases

Supplementary Figure 5 shows the proportion of the body constituents. Within the qi deficiency, Chapter II (Neoplasms) diagnoses were more likely associated with qi deficiency, which was only seen in the male group. Within the kidney qi deficiency, Chapter XIII (Diseases of the musculoskeletal system and connective tissue) was more likely associated with kidney qi deficiency in males and females. Interestingly, chapter XIV (Diseases of the genitourinary system) was less likely associated with kidney qi deficiency when taken as a whole. However, in the male subgroup, chapter XIV is positively associated with kidney qi deficiency, which was not seen in the female subgroup. Within qi stagnation, chapter V (Mental and behavioral disorders) diagnoses were more likely to be associated both in males and females.
